# Pulmonary Complications of HIV

**DOI:** 10.3201/eid2107.150500

**Published:** 2015-07

**Authors:** Sushma Cribbs

**Affiliations:** Emory University School of Medicine, Atlanta, Georgia, USA; Department of Veterans Affairs, Atlanta

**Keywords:** HIV, human immunodeficiency virus, viruses, lungs, pulmonary complications

Pulmonary Complications of HIV summarizes current practices for diagnosing and treating common HIV-related pulmonary complications. It is a well-written, educational work that will interest anyone managing the care of HIV-infected persons. The content and amount of information packed into this easy-to-read textbook is impressive. Each chapter is well organized and well referenced, and important concepts and definitions are laid out clearly.

Since HIV/AIDS was first described, clinicians have found that the lung is the site most frequently affected and that pulmonary complications are a major cause of illness and death for HIV-infected persons. However, over the years, the discovery and use of antiretroviral therapy has increased life expectancy for HIV-infected persons, and the spectrum of infectious and noninfectious pulmonary complications has changed. For example, the incidence of opportunistic pneumonias has declined dramatically, whereas the incidence of bacterial pneumonia has not decreased proportionately. Furthermore, noninfectious complications, such as chronic obstructive pulmonary disease and lung cancer, are increasing. It is critical for anyone managing the care of HIV-infected persons to be aware of these lung complications and understand their diagnoses, possible treatments, and prevention. Pulmonary Complications of HIV does an excellent job discussing these aspects.

The authors are well-respected researchers and clinicians from throughout the world who work in the fields of pulmonary medicine and HIV-related lung diseases. The literature on HIV-related pulmonary complications is still lacking in certain areas, which most likely led to some chapters (e.g., Bronchiectasis) to be shorter and less comprehensive than others. Of the book’s 19 chapters, the first 2 discuss the global epidemiology of HIV and current antiretroviral therapy guidelines, which will be useful for clinicians who might not regularly manage the care of HIV-infected patients. The third chapter discusses pulmonary immunity, a complicated topic but one the authors explain simply by emphasizing essential concepts. The next several chapters highlight various diseases and strategies for preventing them in the field, including vaccine guidelines. In addition, the authors cover a number of other key aspects to HIV care, such as pregnancy, pediatrics, and infectious and noninfectious complications, completing a thorough review of the literature. Although infectious disease specialists and others who care for HIV-infected patients might consider the first few chapters too simplistic, the latter chapters on pulmonary complications will be relevant and instructive. Pulmonologists will find that the first few chapters discuss aspects of HIV care to which they are not readily exposed and the latter chapters provide information on the epidemiology, clinical manifestations, diagnosis, and management of common complications seen in HIV-infected persons.

Pulmonary Complications of HIV does an exceptional job summarizing the major pulmonary manifestations of HIV/AIDS and discussing the progress in overall HIV treatment. Because the book itself is fairly short (265 pages), it appears to be more of a simple paperback rather than a reference textbook. Regardless, it is worthy of a spot on your bookshelf. As a clinician, educator, and researcher in the field of HIV-related lung disease, I found the book to be informative, easy to read, and a quick and simple reference to have on hand. It would be valuable to any medical trainee or clinician who manages the care of HIV-infected patients.

